# First findings of brown hare (*Lepus europaeus*) reintroduction in relation to seasonal impact

**DOI:** 10.1371/journal.pone.0205078

**Published:** 2018-10-10

**Authors:** Jan Cukor, František Havránek, Rostislav Linda, Karel Bukovjan, Michael Scott Painter, Vlastimil Hart

**Affiliations:** 1 Forestry and Game Management Research Institute, Jíloviště, Czech Republic; 2 Czech University of Life Sciences Prague, Faculty of Forestry and Wood Sciences, Department of Silviculture, Suchdol, Czech Republic; 3 Czech University of Life Sciences Prague, Faculty of Forestry and Wood Sciences, Department of Game Management and Wildlife Biology, Suchdol, Czech Republic; Sichuan University, CHINA

## Abstract

In Europe, brown hare (*Lepus europaeus*) populations have been declining steadily since the 1970s. Gamekeepers can help to support brown hare wild populations by releasing cage-reared hares into the wild. Survival rates of cage-reared hares has been investigated in previous studies, however, survival times in relation to seasonality, which likely plays a crucial role for the efficacy of this management strategy, has not been evaluated. Here we examine the survival duration and daytime home ranges of 22 hares released and radio-tracked during different periods of the year in East Bohemia, Czech Republic. The majority of hares (82%) died within the first six months after release, and 41% individuals died within the first 10 days. Significant differences were found in the duration of survival with respect to the release date. Hares released in the summer months (July and August) survived the longest (on average 103.2 days, SD ± 23.8) and hares released throughout all other months of the year survived for significantly shorter periods of time (on average 20.4 days, SD ± 11.5). The most likely cause of death was red fox predation (38.9%) followed by disease (coccidiosis and other health problems) (27.8%). Three hares (16.6%) were killed by automobile traffic. After six months of radiotracking, we found the average survival time of all hares released was 58 (SD ± 70.9) days. Hares in this study preferred to remain in the vicinity of the release area and the average distance from release point to the center of the home range was 471 m.

## Introduction

The decline in the number of small game is documented by numerous studies across Europe [1,2]. However, the brown hare (*Lepus europaeus* Pallas 1778), despite the declining population, is still one of the most widespread game species found in Central Europe [3]. The hare population began to decline significantly during the 1970s [4–9] and some researchers report a wider range of population decline in the last few decades of the twentieth century [3,10,11]. The cause of the decline is often attributed to the intensification of agricultural production and dramatic declines in the diversity of agricultural crops [12–14] as well as the diversity of native flora [15,16].

In heavily farmed landscapes, it is very difficult for brown hare to find suitable shelter, not only from predators, but also to provide refuges from adverse climatic conditions. This is especially true for juveniles [17,18] where the absence of suitable shelters can impact important physiological processes, such as thermoregulation, and result in negative energy balances [17]. The increasing use of paraquats, a commonly applied and readily available agricultural pesticide, was analysed by Edwards [4], however his conclusions show that paraquats are not a significant cause of hare deaths. Changes in food supply, such as limited plant diversity as a result of monoculture farming practices, also influence the brown hare population. Indeed, hares have been shown to prefer heterogeneous habitats with variable and diverse food resources [19,20].

Among other things, the shift in farming practices has also been shown to increase the home range size of hares [21,22], creating another factor that influences their survival rate in the current cultural landscape. Increased home ranges force hares to expend more energy [13,23,24] and increases the risk of predation [13,25]. However, there are many causes of hare mortality, most noticeable is predation by the red fox (*Vulpes vulpes*) [26–30] as well as disease, climate change and collisions with vehicles [8,31,32]. In addition, hares are hunted, not only for sport, but also for their valuable game meat [33].

In Europe, a number of small game populations, including the brown hare, can be supplemented by changes in the landscape, and perhaps most importantly, by changes in agricultural subsidy policies such as those previously proposed [34,35]. The reformation of Common Agricultural Policy includes a “greening program” which is comprised of crop diversification and maintenance of permanent grasslands as well as ecological focus areas [36]. Changes in subsidy policy have been applied slowly and there has been no significant shift in the use of agricultural land with respect to agricultural field sizes and landscape diversity.

To increase hare populations, it is also possible to translocate wild individuals from areas where the population has stabilized or release cage-reared individuals from rearing stations. Initial studies from Italy and Poland testing survival duration of cage-reared individuals have been published [9,37,38]. An overall evaluation, which summarized 25 papers (mostly based on data from Italian populations), addressing different factors including mortality of releasing brown hares has been described by Sokos [39]. Conclusions from this meta-analysis show increased survival rates of cage-reared hares when acclimated to natural conditions by first exposing hares to adaption enclosures. However, survival rates of cage-reared individuals was still relatively low and the survival time relative to release data was not considered. In the Czech Republic, neither survival duration nor performance of cage-reared hares has been studied.

The Ministry of Agriculture in the Czech Republic is also aware of the declining hare population and has decided to support reintroduction efforts, either by catching and translocating wild hares or by cage-rearing methods. However, due to the sparse hare population in the Czech Republic, it is rather difficult to catch wild individuals and, in practice, the release of cage-reared individuals is often preferred. The terms and conditions for the release of wild hares are regulated in § 38, Financial Contribution to Support Endangered Species of Wild Game and Brown Hare, which falls under Government Decree No. 30/2014 on Binding Rules on the Granting of Financial Contributions to Forest Management and Selected Hunting Activities. The allocated amount for a single hare is CZK 1,500 (approximately EUR 60) for the release of a minimum of 10 individuals. Each hare must be permanently marked and each mark registered for five years. Hunting brown hares is not permitted for a five-year period following their release in respective hunting district.

However, the government decree does not regulate the appropriate date nor the method of release, which can be done through adaptation fence plots or directly from transport boxes. The aim of this work is to: (1) evaluate the survival duration of cage-reared hares relative to the time of release, and (2) describe the behaviour of released hares in terms of habitat utilization and size of home ranges during the resting part of the day (hereafter referred to as inactive home range).

## Material and methods

### Study area

Hares were released in the České Meziříčí hunting district between 2015 and 2017. The hunting ground is located 12.8 km northeast of Hradec Králové in eastern part of the Czech Republic. The hunting ground has an average altitude of 260 m a.s.l, with the highest point at Bílý vrch (299 m a.s.l.). Average annual temperature of the broader area of interest (for Hradec Králové) is 8.5°C and the average annual precipitation is 617 mm. The adaptation fence plot used to acclimate and release game was located in the southern part of the hunting ground (GPS: N 50° 14.7', E 14° 2.37'). The total area of the České Meziříčí hunting ground is 1607.4 hectares. The agricultural land occupies 1381.5 ha (86%), forested land covers16.5 ha (1%), water area comprises 37.5 ha (2%) and other areas (e.g. roadways, recreational areas, urban greenery, field roads, etc.) make up 171.9 ha (11%). The most common agricultural crop in this area is sugar beet followed by corn, grain crops and rapeseed. The average size of agricultural fields in the study area was 14.3 hectares. The standard number of hare was 267 (approximately 17 hares/100 ha) and the minimum was 79 (approximately 5 hares/100 ha). In 2017, the spring count was 176 hare (approximately 11 hares/100 ha), which is comparable with population densities in other areas of the Czech Republic [40], but lower than values reported from other European countries (e.g. 28–43 individuals/100 ha [7]).

### Study animals

From 2015–2017 (all radiotracking was completed by December 31, 2017), a total of 22 hare were fitted with radiotelemetric transmitters and released by removing the fencing enclosing the acclimation plot. Released individuals were at least 6 months old. The dates of release and sex can be found in Table 1. Prior to release, sex, health status and weight were recorded and each hare was given a veterinary examination. Male and female average weights were 2.8 kg (± 0.38 kg) and 3.5 kg (± 0.42 kg), respectively. All individuals were obtained from a professional breeder, Jaroslav Horáček, chairman of the Association of Hare Breeders of the Czech Republic where they were kept in a 4 m^2^ covered pen. Prior to their release, hares were fed a complex granulated diet that included a high-quality meadow hay and had access to fresh drinking water ad libitum.

**Table 1:** Release date and sex data for all brown hare involved in the study.

	Release date								
	October 2015	February 2016	July 2016	April 2017	August 2017	October 2017	Summer months	Rest of the year	Total
Males	0	1	1	2	3	2	4	5	9
Females	3	1	2	2	4	1	6	7	13
Total	3	2	3	4	7	3	10	12	22

Hares were released from the adaptation fence plot, enclosed by standard forestry fencing (fence perimeter: 182 m, total height of fence: 160 cm, wire eyes were about 5**×**15 cm) that provided protection from predators and was fortified to prevent small game from escaping. The total area of the adaptation plot was approximately 1300 m^2^. One-third of the adaptation area was occupied by Jerusalem artichoke (*Helianthnus tuberosus*), which provided cover and food options. Hares were also supplemented with fresh water and granulated feed. When releasing hares, the wire-netting was removed from the adaptation fence, allowing hare to freely roam outside the adaptation area and also return to the area after dispersing. Adaptation was performed in 2-week periods, to provide the hares with sufficient time to become accustomed to their new surroundings. Prior to entering the adaptation fence plot area, hares lived only in the metal grate of the aviary in which they were born and bred.

### Radiotracking

Prior to entering the adaptation fence plot, each hare was equipped with a radiotracking collar equipped with a TW 3 1/2AA telemetry transmitter (Biotrack, United Kingdom) with an approximate weight of 25 grams and guaranteed life span of 13 months. Transmitters were equipped with a mortality sensor, which was activated if the collar remained motionless for more than a 24 hour period. Transmitter frequency ranged between 150.000 to 152.000 MHz, with accuracy resolution up to three decimal places. Radio signals could be detected up to 4 kilometres from the individual depending on terrain and climatic conditions. On average, positional data was obtained for each hare once per day, five days per week. Radiotracking was performed only during the day. Individuals were located with the aid of traditional triangulation methods [41,42].

### Data processing

For all hare that died during the study, cause of death was determined. When in doubt, hares were given an autopsy to identify the cause of death. In two such cases, X-rays were needed to confirm that vehicular collisions were the cause of death.

Once radiotracking was complete, the total area of individual home ranges was determined, however, home ranges were analysed only for hares that survived for more than 30 days, as the first month after release was considered as an exploratory period when hares were adapting to, and selecting suitable habitat within the novel landscape. To evaluate the size of the inactive home ranges, kernel density estimations (KDE) were computed via R package “rhr” [43] and 95% kernel density contours were selected as ‘inactive home ranges’, while the remaining 5% of locations were taken as extremes representing escape responses of disturbed hares. Data was processed in ArcGIS 10.5 (ESRI). The Orthophoto dataset, provided by the Czech Land Survey Office (ČÚZK), was used as the underlying map.

### Statistical analysis

The Kruskal-Wallis test was used to test for differences between the mean survival time as a function of the cause of death, and subsequent multiple comparisons. The dependence of the mean survival time with respect to the release period (summer vs. other) was tested using the Wilcoxon rank-sum test, as well as the differences in mean survival times between males and females.

Home range sizes were also compared between males and females using the Wilcoxon rank-sum test. The relative area of fields and of meadows within the total home range area was tested by regression analyses.

To evaluate survival time, daily mortality was computed, as described by Heisey and Fuller [44], using the following equation: *DM* = 1 –(1 –*TM*)^1/*d*^, where *DM* = daily mortality, *TM* = total mortality (to the nearest decimal) and *d* = duration of evaluated period in days, and therefore, can be used to evaluate daily mortality across different test durations. All statistical analyses were performed using α = 0.05 level of significance. Computations were performed in R software [45].

## Results

### Causes of hare mortality

Over the entire study period, the mortality rate was 82%, with only four hares surviving more than 6 months. Nine hares (41%) were found dead within the first 10 days after release from the adaptation fence plot, with a total of 12 hares (54.5%, includes the 9 aforementioned hares) deceased within the first 30 days after release. The main cause of death was predation by red foxes (38.9%) (Table 2). Five hares (27.8%) died of health problems and the final cause of death was determined by an autopsy. Autopsies reported two cases of stomach inflammation, two cases of enlarged adrenal glands due to stress, and one case of myocardial haemorrhage was detected. In three cases, the cause of death was due to collisions with automobile traffic. In two cases, cadavers were found in an advanced stage of decomposition. We speculate that this was due to a delay in the activation of the mortality sensor probably due to a predator moving the cadavers preventing the mortality sensor to activate. Only one signal was lost and the corresponding transmitter was not found. A possibility for this circumstance could be destruction by farming machinery. Indeed, the signal was lost on a day when the meadow was being mowed. The average hare survival duration was 58 days (SD ± 70.9), which included the four hares that were still alive at the end of the monitoring period, and whose survival duration (182 days) increased the overall average survival time of all released hares.

**Table 2:** Causes of hare mortality.

	*n*	%
Foxes	7	38.9
Health problems	5	27.8
Road traffic	3	16.6
Unidentified	2	11.1
Lost (transmitter could not be found)	1	5.6
*Total*	*18*	*100*

**Fig 1:**
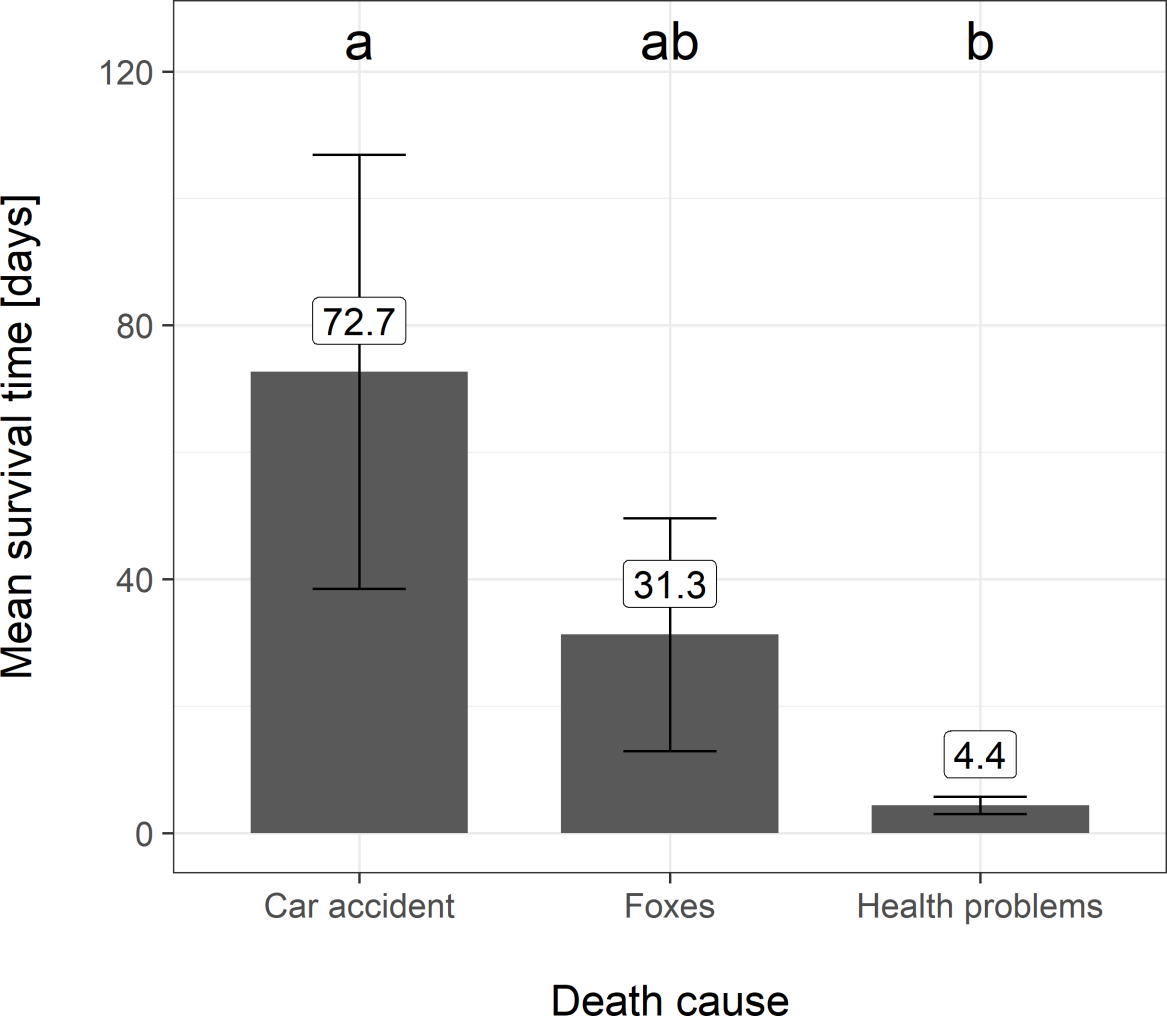
Statistical comparison of the mean survival duration of released hares with respect to the cause of death. The numerical values above each bar show mean values for each cause of death, and lettered indexes above the bars indicate statistical. Error bars depict mean ± standard error.

A more detailed analyses of hare mortality show significant differences between survival duration of released hares with respect to the cause of death (Fig 1); this characteristic was tested by the Kruskal-Wallis test (*p* = 0.03). We found no statistically significant differences between the survival time of males (67.6 days, SD ± 66.9) and females (51.5 days, SD ± 75.5). Survival times were tested using the Wilcoxon test (*p* = 0.25).

**Fig 2:**
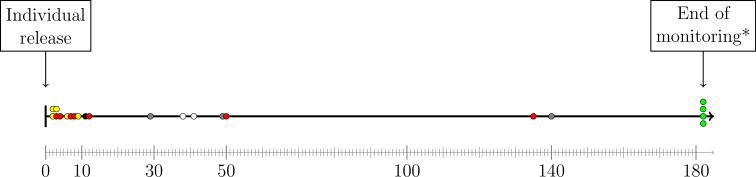
**Survival duration of hares according to cause of death:** *red*–predation by red foxes, *yellow*–health problems, *black*–lost transmitter, *gray –* hit by car, *white*–unidentified, *green*–survived monitoring period. Gray axis below depicts days from individual release.

Hares killed by vehicles had survived 72.7 (SD ± 59.2) days on average. In contrast, hares with health related causes of death died within the first 10 days of release (4.4 days, SD ± 3.05). Hares predated by red foxes had an average survival time of 31.3 days (SD ± 48.6). The detailed distribution of survival duration of hares with respect to release period is shown in the timeline (Fig 2). Hare survival likelihood is significantly increased beyond 20 days after release.

**Fig 3:**
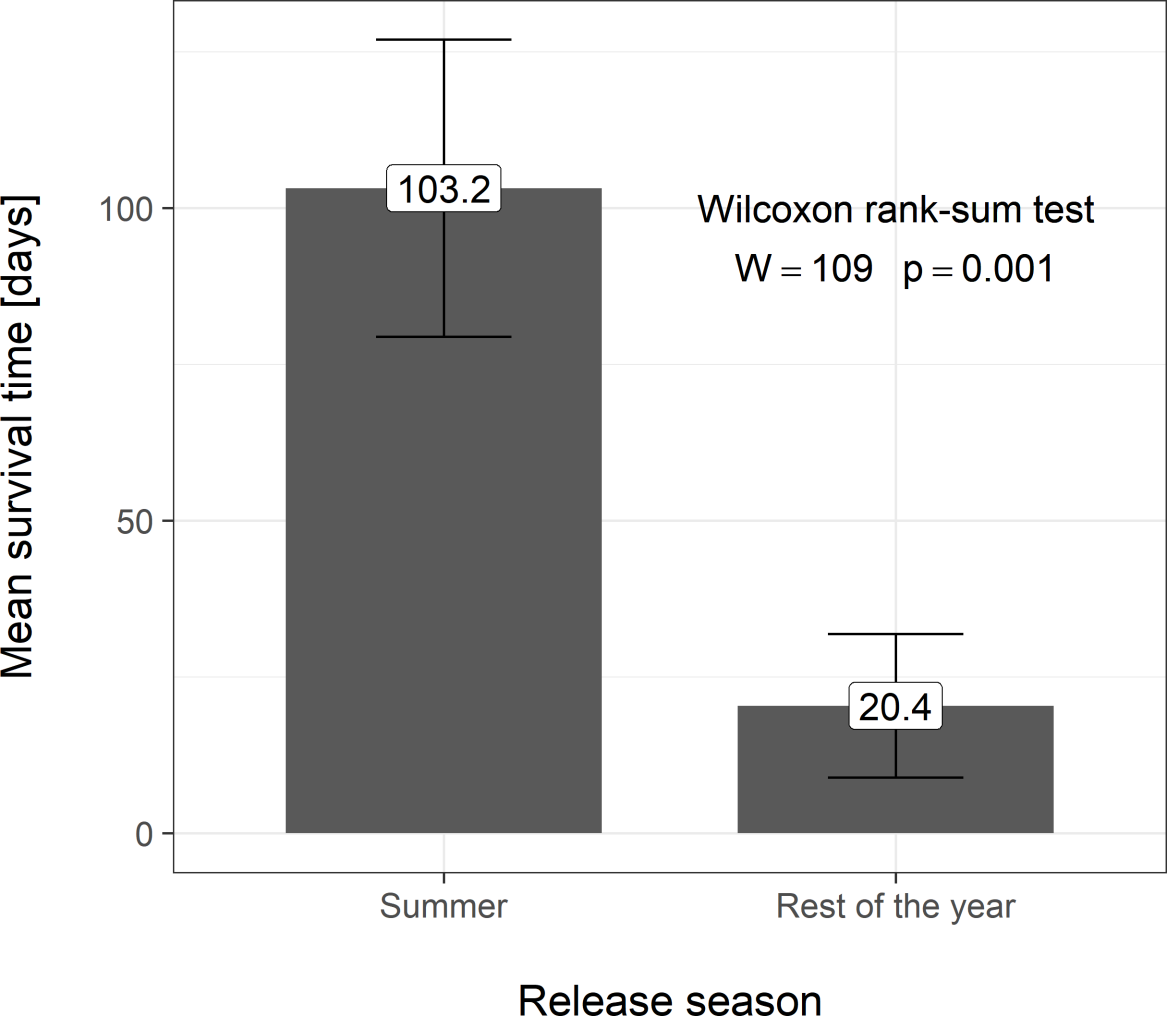
Survival days in relation to the release date of brown hare. The labels above the bars show the mean value of each variant. Error bars depict mean ± standard error.

The survival duration relative to the release period was evaluated. A statistical comparison by the Kruskal-Wallis test (*p* = 0.001) revealed significant differences between the survival duration of hares released in summer months (July and August) (103.2 days, SD ± 23.8) compared to hares released during other months of the year (20.4 days, SD ± 11.5). Comparison of the survival time is evident from Fig 3.

### Home range analysis

Home ranges were described only for hares surviving more than 30 days; shorter survival periods were excluded from home range analysis as we consider these short periods only as adaptation to their natural habitat without time to establish home range areas. As a result, home range size was evaluated for 10 individuals. The average size, based on 68 fixes for each home range, of inactive home ranges (i.e. home ranges calculated from positional data collected during the daytime, therefore, representing the ‘inactive’ period of hare activity cycle) was 15.7 ha (SD ±14.6). The average inactive home range averaged 18.8 ha (SD ± 18.4) for males (*n* = 5) and 12.5 ha (SD ± 10.9) for females (*n* = 5). and was not significantly different between sexes (Wilcoxon test, *p* = 0.55). The average distance from adaptation plot to the center of the daytime home range was 471 m across all hares.

**Fig 4:**
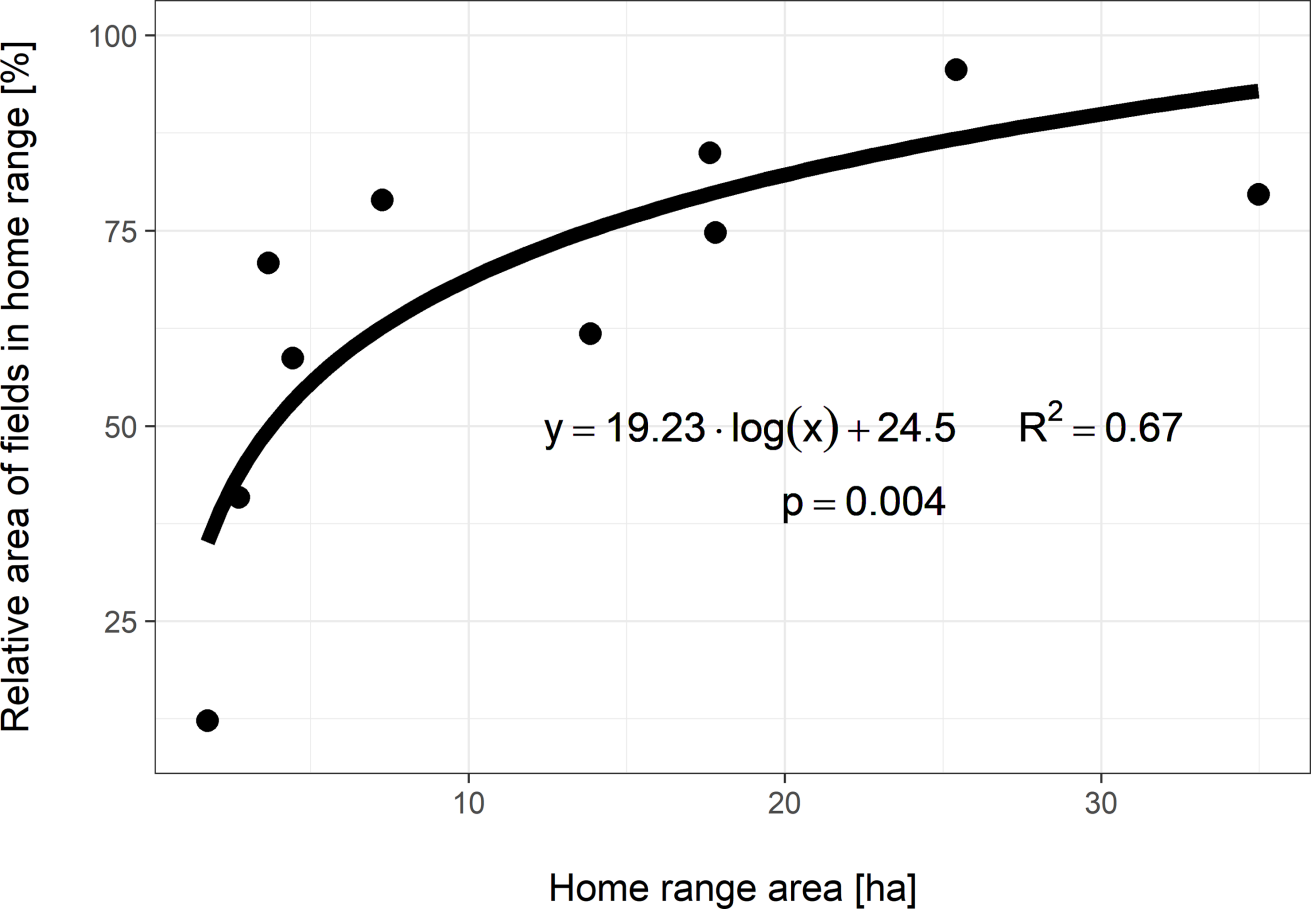
Agricultural field area percentage relative to inactive home range size. Bold line depicts regression function described in the plot. *P*-values for logarithmic regression assume a null hypothesis based on parameter *a* of the regression equation *y* = *a* * *log*(*x*) + *b* is equal to zero. Natural logarithm was used for regression function.

**Fig 5:**
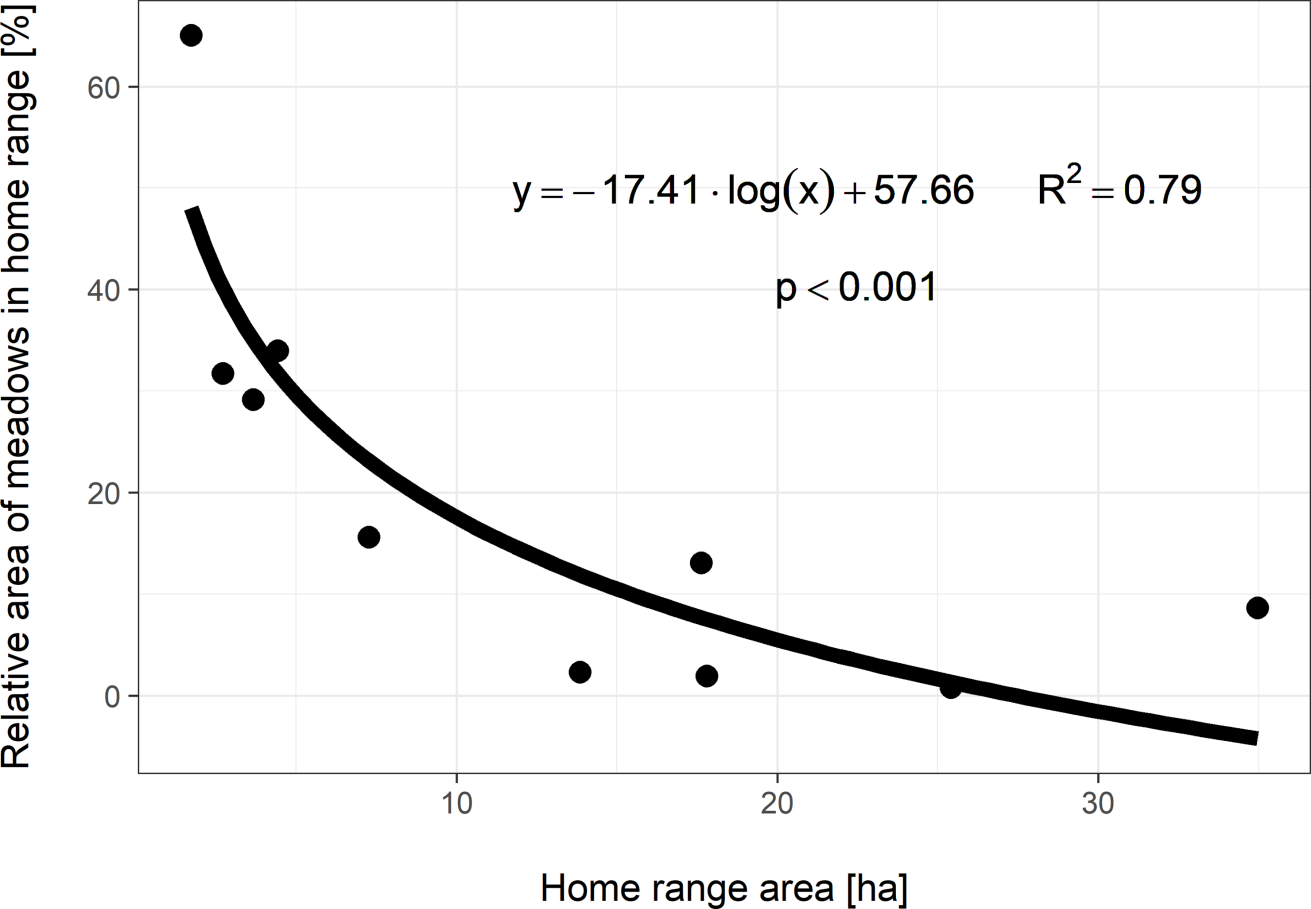
Meadow area percentage relative to the size of inactive home ranges. Bold line depicts regression functions described in the plot. Provided *p*-values for logarithmic regression assume a null hypothesis based on parameter *a* of the regression equation *y* = *a* * *log*(*x*) + *b* is equal to zero. Natural logarithm was used for regression function.

Home ranges were further evaluated based on habitat characteristics. The relationship between the proportion of agricultural field area relative to inactive home range sizes was analysed by logarithmic regression analysis. Fig 4 shows a clear, positive correlation between percent of agricultural field area and inactive home range size (*p* = 0.03). Alternatively, analysis of the proportion of meadow vegetation shows a significant negative correlation relative to total inactive home range area (*p* = 0.04) (Fig 5). The *p*-values for logarithmic regression assume a null hypothesis based on parameter *a* of the regression equation *y* = *a* * *log*(*x*) + *b* is equal to zero. Natural logarithm was used in both regression analyses.

## Discussion

The aim of this work was to determine the survival duration and causes of mortality in released cage-reared hares. In general, a relatively low survival rate was expected within the first days after release, when cage-reared hares were the most vulnerable to unfamiliar surroundings. Similar mortality rates were described for cage-reared hares in Greece [46]. After the first 10 days, 43.8% of the released hares had died. A higher survival rate of cage-reared individuals was recorded in Poland, where 38% of hares died within the first 30 days after release [9]. In Italy [37], 44 released hares (68%) died within the first 10 days after release, however, these individuals were wild-caught and relocated from places containing high-density hare populations.

Hare mortality was also studied by Sokos [39], who analysed 15 publications on cage-reared hare survival rate (mostly without previous adaptation). This analysis showed that 60% - 90% of hares died within the first month of release. It is also possible to compare mortality rate by daily mortality in percentages. Our results indicate a daily mortality rate of 1.1% for August (30-day evaluation) and 14.5% for October (7-day evaluation). Comparable data from cage-reared hares released with previous adaptation were published in Italy (European hares) and Sweden (mountain hares). The daily mortality rate recorded in August was 5.5% [47] and 1.9% [48]. In December, a 15.3% daily mortality rate was found in Dematteis *et al*. [48], and are consistent with our findings. Although the release date of caged hares with prior adaptation was mentioned in previous studies, it has not been independently evaluated in any publication to date.

We observed a very high overall mortality rate in brown hare, with only 18% surviving the entire study period after release from the adaptation fence plot. Released hares surviving for more than six months were considered individuals who became adapted to natural conditions. In accordance with other authors [37,39,46,48], this statement supports our results which shows rapidly increasing survival rates due to adaptation to natural conditions after the first 30 days from the initial release date. The high mortality rate recorded in our study, and perhaps previous studies, could be influenced by intentionally releasing hares in different periods of the year. We suspect that survival duration of released hares would have significantly increased if the release dates occurred only in the summer season.

The release period appeared to play a crucial role in the successful reintroduction of caged hares with previous adaptation, as survival time is significantly related to release period. The longest survival rate was recorded in July and August (103.2 days, SD ± 23.8) compared to months evaluated from the remainder of the year (20.4 days, SD ± 11.5; February, April and October). In the summer months, food resources, as well as suitable cover options to shelter from predators and from adverse weather were more abundant, and likely contributes to survival success during hare releases during this period. Indeed, this explanation is consistent with findings from wild hare populations [13,19].

However, the effects of climate variables on hare population dynamics are complex and temporally dependent, and therefore, cannot be overlooked [7]. Temperature has been shown to be positively correlated with hare abundance [7,49], and increasing winter temperatures across most of the globe are correlated with reduced snow cover [50,51], reducing hare energy expenditure [17]. Furthermore, while shifts in climate resulting in decreased precipitation and increased winter temperature have been shown to decrease mortality rates of leverets, particularly within the first two weeks after birth [7,17], low precipitation in the summer has been shown to decrease population sizes of adult hare [17,52]. While the population effects of climate change on wild hare are undoubtedly complex, seasonally variable, and likely age-dependent, increasing temperatures [50,51,53] and shifts in precipitation patterns are likely to impact hare population dynamics, and therefore, should be considered in hare population management plans.

A key factor influencing survival rate of released hares with previous adaptation could be that individuals with more experience and familiarity with their surroundings are able to survive for longer periods of time (i.e. evade predators, efficiently locate valuable resources). Indeed, our data suggest that after a period of approximately 15 days, hares are much more likely to evade predation and starvation. Generally, wild animals have greater chances to create cognitive maps, which then facilitates field orientation and provides them with a capacity to return to preferable locations from longer distances [54,55]. However, it is not yet known how much time (since individual release) caged hares need to develop a functional cognitive map.

The most common cause of hare mortality was predation by red foxes, and is consistent with data from previous studies evaluating survival durations of cage-reared hares after release. Average red fox predation rates were found to fall between 28.6 to 43.7% [37,38,46], and therefore, our results are fully in line with the findings from previous studies. Red foxes have been shown to be the predominant predator of both juvenile and adult wild hares [4,56–62]. Fox predation intensity is often expressed as the percent of whole-year hare production predated upon by red foxes. These values are highly variable and are influenced by red fox and hare population densities within a given area [29]. Most studies report red fox predation rates between 10–40% on wild hares [56,63] however, some studies have reported predation rates of up to 100% for juveniles [57]. Hare predation by red foxes is also possible to evaluate from assessing fox stomach content. The proportion of hare biomass in the red fox stomach was about 40% in years with high hare population densities [56,61]. Results from the last twenty years show a significantly lower proportion of hare biomass in red fox stomach content ranging from 1–13% according to locality and hare population densities [64–67]. Red fox predation is also influenced by environmental factors, for example, severe winters with high snow accumulation can account for up to 50% of the total hare mortality [56].

The telemetry transmitter could possibly affect the risk of predation by large predators like the red fox. Although effects of animal telemetry tags, such as those used in the current study, have not been conclusively demonstrated, many researchers have assumed that hares are much more vulnerable to predation in the first week of carrying transmitters [68–70]. However, our intent was to acclimate hares to the telemetry collars during the two week adaptation period, and therefore, reduce the confounding variables related to telemetric collars and predation. Furthermore, the relatively low weight of the transmitter (25 g), which is less than one percent of the individuals’ body mass is thought to have limited impact on hare mobility and body condition [68]. Heavier GPS collars (70 g) [24], or VHF transmitters with added functions like motion-sensitive radio-transmitters, typically weighing approximately 3–4% of the total body mass of an adult hare may have greater influence on hare behaviour [71].

Another cause of hare mortality were health-related problems that developed within the first ten days after release. It is assumed that hares died due to a combination of factors, such as post-release stress, starvation, coccidiosis and other diseases. For intensively reared hares, coccidiosis and other diseases present a significant health risk to the animal, and may lead to death, particularly shortly after release. Death due to coccidiosis and other diseases have also been reported by Angelici [37] in 10.6% of individuals. Therefore, we recommend administering anticoccidiosis vaccinations before releasing hares into adaptation or wild areas. Furthermore, diseases usually associated with wild European hare mortality are not usually found in cage-reared and released hares. Diseases typically reported in wild hares are tularaemia, brucellosis and also European Brown Hare Syndrome virus [72–74], however, antibodies against tularaemia and brucellosis were found in the blood sera of hunted hares in small quantities not thought to pose a series health risk [72–76]. Therefore, the available evidence suggests that diseases play a rather limited role in wild European brown hare populations [77] and may help to explain why cage-reared and released hares are at seemingly low risk for diseases beyond the first few days after release.

The third most frequent cause of death in hares was collisions with road traffic. Traffic collisions of cage-reared and released hares was also studied in Poland, ranging in mortality rates from 4.7 to 7% [9,38]. To some degree, this risk can be mitigated by selecting suitable release sites with sparse traffic in the vicinity of the adaptation area. However, landscape fragmentation by roadways is ever increasing [5] and traffic statistics show negative impacts on automotive use and hare population sizes, with hares being the most common victim of automobile-related deaths, compared to other animals, in Central Europe [8,32,78] Fortunately, there has been a gradual improvement in high-traffic motorways with the instillation of barrier fences, warning signs and/or modifications of habitats along the roadways to protect against wildlife collisions [79,80].

As discussed above, home range size was calculated from data collected during the day, when hares are relatively inactive. Not surprisingly, these ranges are much smaller compared to total home range sizes which include locations for both inactive and active times of the day and have been described for cage-reared hares (e.g. in Poland, the average home range size was as large as 130 ha) [9]. We did not find significant differences in the size of inactive home ranges between males and females in our study.

Released hares were inclined to stay in the areas near the adaptation fence plot area. Similar spatial behaviours were also found in translocated wild hares, 71% of whom travelled no farther than 800 m from the release site [37]. The size of the daytime home range increased in relation to the percentage of agricultural fields, e.g., when the fields comprised more than 80% of the home range, the size of the home range area increased to more than 25 hectares. Indeed, in wild hares, a positive correlation between home range size the size of agricultural field plots, is often found [19,81–84].

## Conclusion

Our results highlight the impact of release period (month) on survival duration. Release time has been mentioned in previous studies, but seasonality has not been evaluated with respect to mortality and survival duration.

Survival duration significantly increased when hares were released in the summer months (July and August), when essential resources, such as food and shelter options, are abundant. Cause of death was most often attributed to predation by red foxes and the highest mortality rate occurring within the first ten days after release from the adaptation fence plot (41% of the individuals). Released hares tended to remain in the vicinity of the release site, and therefore, home ranges overlapped, or were in close proximity to the adaptation plot and where hares first encountered the natural and free-ranging conditions for the first time. Therefore, management of wild brown hare populations using cage-rearing and release methods can be optimized by releasing hares during the summer months, selecting release sites containing suitable shelters, and administrating anticoccidiosis vaccinations prior to release. These results shed a new light on the possibilities of releasing previously adapted, cage-reared hares with respect to release date that could support wild European hare populations in areas with low abundance or declining numbers in Central Europe.

## Supporting information

S1 Table Dataset used for statistical analyses of home ranges and mortality of studied hares. (CSV)
